# PRL3 phosphatase active site is required for binding the putative magnesium transporter CNNM3

**DOI:** 10.1038/s41598-017-00147-2

**Published:** 2017-03-03

**Authors:** Huizhi Zhang, Guennadi Kozlov, Xinlu Li, Howie Wu, Irina Gulerez, Kalle Gehring

**Affiliations:** 1 Department of Biochemistry, McGill University, Montreal, Quebec Canada; 2 School of Chemical Engineering, Northwest University, Xi’an, 710069 China

## Abstract

The phosphatases of regenerating liver (PRLs) are involved in tumorigenesis and metastatic cancer yet their cellular function remains unclear. Recent reports have shown PRL phosphatases bind tightly to the CNNM family of membrane proteins to regulate magnesium efflux. Here, we characterize the interactions between the CBS-pair (Bateman) domain of CNNM3 and either PRL2 or PRL3 using X-ray crystallography, isothermal titration calorimetry, and activity assays. We report four new crystal structures of PRL proteins bound to the CNNM3 CBS-pair domain that reveal the effects of cysteine disulphide formation and nucleotide binding on complex formation. We use comprehensive mutagenesis of the PRL3 catalytic site to quantify the importance of different PRL amino acids, including cysteine 104, leucine 108, and arginine 110, for CNNM binding and phosphatase activity. We show the PRL3 R138E mutant is selectively deficient in CNNM3 binding with the potential to distinguish between the downstream effects of phosphatase and CNNM-binding activities *in vivo*. Through a novel activity assay, we show that PRL3 has magnesium-sensitive phosphatase activity with ATP and other nucleotides. Our results identify a strong correlation between phosphatase activity and CNNM binding and support the contention that PRL function as pseudophosphatases regulated by chemical modifications of their catalytic cysteine.

## Introduction

The phosphatases of regenerating liver (PRLs) are highly over-expressed in metastatic cancers yet their mechanism of action is poorly understood^1, 2^. Like other members of the family of protein tyrosine phosphatases, their phosphatase activity occurs through a two-step catalytic cycle involving the transient phosphorylation of a catalytic cysteine residue^3^. In PRL phosphatases, this intermediate is extremely long-lived leading to the accumulation of a cysteine-phosphorylated form of the enzyme both *in vitro* and in cells^4, 5^. While a number of different cellular substrates have been proposed, there is no consensus about their physiological substrate due, in part, to their slow rate of overall catalysis^2^. The catalytic cysteine of PRLs readily forms a disulfide with the adjacent cysteine residue, which further decreases their effectiveness in dephosphorylating physiological substrates^4, 6^.

A major breakthrough occurred two years ago with the identification of CNNM proteins, a family of membrane proteins involved in magnesium homeostasis, as PRL-binding partners^7, 8^. Disruption of the PRL-CNNM interaction promotes tumor formation and invasiveness in animal and cellular models, strongly suggesting that the physiological function of PRLs is to regulate CNNM magnesium transport. Identification of the CBS-pair domain in CNNM proteins as the interaction site led to the first crystal structure between PRL2 bound to the CBS-pair domain of CNNM3^5^. That structure showed the unphosphorylated form of PRL2 bound to the CBS-pair domain of CNNM3 via an extended loop that contacts the PRL active site. Mutagenesis showed that a CNNM3 aspartic acid residue was required for high affinity binding, suggesting that the CNNM CBS-pair domain might act as a pseudo-substrate. Addition of the CBS-pair domain inhibited phosphatase activity and CNNM3 binding was blocked by phosphorylation of the PRL2 active site cysteine^5^. While this manuscript was in preparation, the structure of PRL1 bound to the CBS-pair domain of CNNM2 was released^9^.

Here, we report four new structures of PRL3 or PRL2 bound to the CBS-pair domain of CNNM3. The structures reveal why disulfide formation dramatically decreases binding affinity and confirm that all three PRLs bind to CNNMs. We use isothermal titration calorimetry (ITC) experiments and extensive mutagenesis to probe the importance of PRL3 residues for CNNM binding. Comparison of binding activity and *in vitro* phosphatase activity shows that they are strongly correlated with the notable exception of the PRL3 R138E mutant which showed weak CNNM3 binding but normal phosphatase activity. Finally, we show that inclubation with a wide variety of cellular phosphate-containing compounds leads to significant PRL cysteine phosphorylation, explaining the phosphorylation of PRLs when expressed in bacteria. These results support the hypothesis that PRLs function as pseudophosphatases in regulating the action of CNNM proteins in cancer.

## Results

### Crystal structures

**Figure 1:**
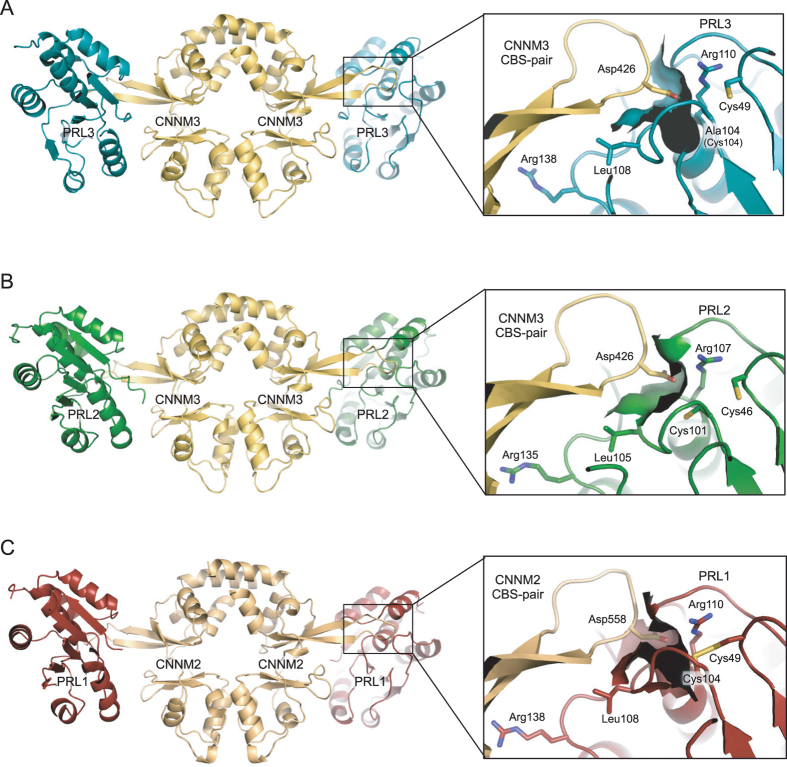
PRL3 complex with CBS-pair domain from CNNM3 compared with previous structures with PRL2 and PRL1. The complexes are similar overall but have significant differences in the catalytic pockets. In all the complexes, the CBS-pair domain forms a central homodimer with an extended loop from each domain that binds PRLs. A CNNM aspartic acid residue inserts into the phosphatase active site and is essential for complex formation^5^. (**A**) PRL3•CNNM3 complex. The PRL3 catalytic cysteine 104 has been mutated to alanine to prevent disulfide formation with cysteine 49. The mutation opens a deep pocket in the catalytic site. (**B**) PRL2 complex with CBS-pair domain from CNNM3 (PDB 5K22) from Gulerez *et al*.^5^. The catalytic cysteine contacts the CNNM aspartic acid effectively closing the substrate pocket. (**C**) PRL1 complex with CBS-pair domain from CNNM2 (PDB 5MMZ) from Gimenez-Mascarell *et al*.^9^. The catalytic cysteine 104 is oxidized as a disulfide. This shifts the cysteine and opens up a pocket similar to that observed in the PRL3 (C104A) complex.

We crystallized PRL3 C104A with the fragment of CNNM3 that had previously been used to study the complex with PRL2^5^. The catalytic cysteine was mutated to alanine to avoid its oxidation to form a disulfide with a neighboring cysteine^4^. As observed in previous PRL•CNNM complexes, the CBS-pair domain forms a homodimer in a head-to-head orientation with each CBS-pair domain binding one phosphatase molecule^5, 9^. In the PRL3 crystals, there are two copies of the PRL3•CNNM3 CBS-pair domain complex in the asymmetric unit (Fig. 1A, Suppl. Table S1). We also crystallized PRL2 with CNNM3 under a variety of conditions in order to assess variability in the complexes. PRL phosphatases are highly prone to oxidation through formation of an intramolecular disulfide bond, Cys101-Cys46 in PRL2, or Cys104-Cys49 in PRL3^4^. The oxidized form of PRL2 readily crystallized with the CNNM3 CBS-pair domain and we were able to obtain 3 crystal structures: two with slightly different CNNM3 constructs and one in the presence of ADP (Suppl. Table S1). Including copies due to non-crystallographic symmetry, we report six new structures of PRL•CNNM complexes which more than doubles the number of structures determined.

Alignment of the complexes shows that all three PRL proteins bind CNNM CBS-pair domains similarly (Fig. 1). The figure also allows comparison of the effects of modifications of the PRL catalytic cysteine: mutated to alanine in PRL3, reduced as a thiol in PRL2^5^, and oxidized to a disulfide in the PRL1^9^. In the complexes, an extended loop from the CBS-pair domains inserts an aspartic acid residue into the PRL active site. The aspartic acid residue is essential for high affinity binding^5^. The phosphatase residues that interact with CNNM proteins are highly conserved across the different PRL isoforms. Within the phosphatase active site, arginine 110 (numbered as in PRL3) provides the positive charge necessary for binding the CNNM aspartic acid residue. The catalytic cysteine plays an important role in modulating the affinity through direct contacts and restructuring of the binding site. Outside of the active site, arginine 138 and leucine 108 mediate conserved electrostatic and hydrophobic interactions.

**Figure 2:**
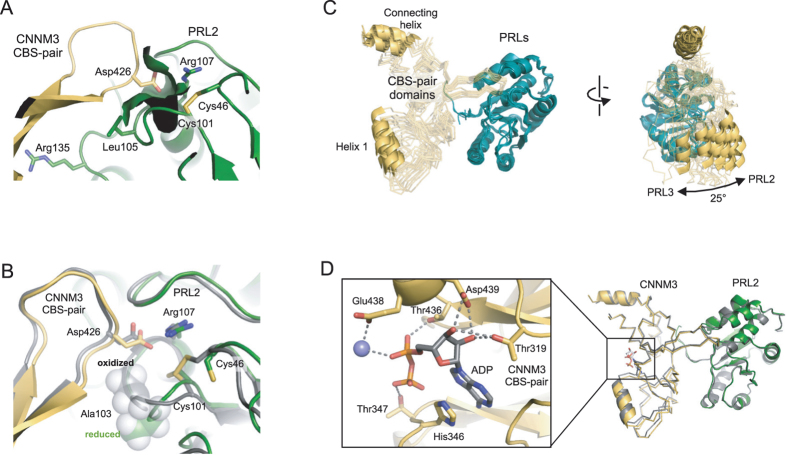
Structural changes upon PRL oxidation and ADP binding. (**A**) Structure of oxidized PRL2 with CNNM3 CBS-pair domain shows formation of the disulfide opens up the large pocket observed in the PRL3 C104A and PRL1 structures. (**B**) Comparison of the reduced (5K22, *color*) and oxidized (this work, *grey*) PRL2 complexes. Upon oxidation, the catalytic cysteine, Cys101, moves away from the CNNM3 aspartic acid and alanine 103 flips to displace CNNM3 by 1 Å. (**C**) Interdomain movement in PRL•CBS-pair domain complexes. Superposition of PRL domains in ten complexes shows rotation around an axis centered on CBS-pair connecting helix. The PDB coordinates compared are 5TSR (two structures of PRL3•CNNM3), 5K22 (reduced PRL2•CNNM3), 5K23 (oxidized PRL2•CNNM3), 5K24 (two structures of oxidized PRL2•mouse CNNM3), 5K25 (oxidized PRL2•CNNM3 with ADP bound), 5MMZ (oxidized PR1•CNNM2, and 5LXQ (two structures of oxidized PR1•CNNM2 with ATP bound). (**D**) Binding of ADP to the CNNM3 CBS-pair domain is mediated by a network of hydrogen bonds. Superposition of the nucleotide-bound (this work, *color*) and unbound (this work, *grey*) structures does not show any significant conformational changes at the PRL2-CNNM3 interface.

We compared the structure of PRL2 with a disulfide to our previously reported structure with reduced PRL2^5^. Oxidation did not induce any large scale conformational changes: the reduced and oxidized phosphatases overlay within 0.6 Å. The largest changes were adjacent to the cysteine residues that form the disulfide bond (Fig. 2A,B). Disulfide bond formation moves the cysteine away from the CNNM loop and flips alanine 103 so that its methyl group points toward the CNNM3 aspartic acid residue. This shifts the CNNM3 loop by 1 Å, which may contribute to the lower affinity of CNNM3 for oxidized PRL2. ITC experiments showed that oxidation of the PRL2 catalytic cysteine decreases its binding affinity by 200-fold^5^.

Globally, the different PRL•CNNM complexes overlay well but the structures shows a shift of up to 20 Å in the relative position of the CBS-pair domain. The shift corresponds to a rotation of up to 25° around the binding site (Fig. 2C). The differences appear to be due to contacts within the crystals rather than intrinsic to the different PRLs or their oxidation status. Non-crystallographic symmetry mates in both the PRL3 (5TSR) and PRL2 (5K24) crystals showed (10°) differences which suggests there is some flexibility in the interaction site that allows the individual molecules to move slightly.

We also crystallized the PRL2•CNNM3 complex in the presence of a mixture of AMP and ATP (Fig. 2D). Electron density was observed at the CBS-pair domain dimer interface but it was intermediate in size and could not be fit satisfactorily by either AMP or ATP. Accordingly, the density was modeled as ADP. While it is possible that ADP was formed by hydrolysis of ATP during crystallization (see below), more likely, the crystals contain a mixture of AMP- and ATP-bound protein. The nucleotide ribose hydrogen bonded with Thr319 and Asp439 while the phosphates associated with Thr347 and Thr436. His346 mediated *π*-stacking interactions with the adenine moiety. A putative metal ion was identified on the dimer axis coordinated by Glu438 and the nucleotide phosphates. This site of nucleotide binding is conserved in other CBS-pair domains including the domain from CNNM2^9, 10^. Other than minor rearrangements, nucleotide binding did not significantly change the conformation of the CBS-pair domain or its interaction with PRL2. Superposition of the free and ADP-bound CBS-pair domains showed a shift of 0.6 Å over 144 atoms. Unlike studies with the free CNNM2 CBS-pair domain, we did not observe a twisting of the dimer interface in the absence of nucleotide^10^.

### Mutagenesis

**Figure 3:**
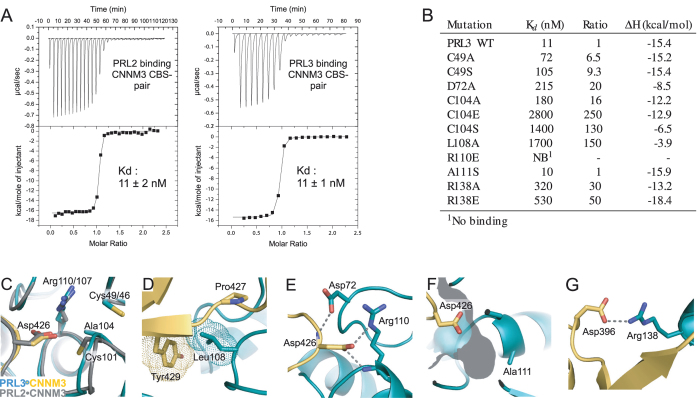
Mutagenesis and affinity measurements identify PRL3 residues required for complex formation. (**A**) ITC measurement of PRL2 and PRL3 binding to CNNM3 CBS-pair domain. The fitting parameters for PRL2 were ${\chi }_{k}^{2}$ 9 × 10^4^, N 1.02 ± 0.003, K_*assoc*_ 8.70 ± 1.5 × 10^7^, ΔH −1.655 ± 0.01 × 10^4^ cal·mol^−1^, ΔS −19.8 cal·mol^−1^·K^−1^. The parameters for PRL3 are given in Suppl. Fig. S1. (**B**) Binding affinity of PRL3 mutants. Selected residues in PRL3 were mutated and the affinity for the CNNM3 CBS-pair domain measured by ITC. (**C**) Complexes of CNNM3 with PRL3 (5TSR, *color*) and PRL2 (5K22, *grey*). Mutation of the PRL3 catalytic cysteine to alanine slightly perturbs the CNNM3 binding site with a modest affect on the binding affinity. Mutations to serine or glutamic acid strongly decrease CNNM3 binding. PRL3 Cys49 is farther from the CNNM3 aspartic acid and its mutation has less effect. (**D**) Hydrophobic contacts of PRL3 Leu108 with CNNM3 Tyr429 and Pro427. (**E**) Key electrostatic contacts between CNNM3 Asp426 and PRL3 Asp72 and Arg110. Loss of Arg110 completely blocks binding. (**F**) Mutation of Ala111 at the base of catalytic pocket does not affect binding. (**F**) Electrostatic interaction between PRL3 Arg138 and CNNM3 Asp396 contributes moderately to binding.

We used the PRL3•CNNM3 complex to test the importance of different phosphatase residues for complex formation. Eleven mutants were prepared and their affinity measured by ITC (Fig. 3, Suppl. Fig. S1). Both PRL2 and PRL3 bind with 11 nM affinity to CNNM3. The apparent affinity is very sensitive to partial oxidation of the phosphatase and the weaker affinity (25 nM) we previously reported for PRL2 was likely due to the presence of a small amount of the disulfide form^4, 5^.

Mutagenesis of the cysteine (Cys49) which forms the disulfide with the catalytic cysteine had a relatively small effect on the affinity (Fig. 3). Cys49 is more than 6 Å away from the nearest CNNM3 residue and the affinity loss was less than 10-fold. Mutagenesis of the catalytic cysteine (Cys104) had a much larger effect. Alanine was relatively tolerated (16-fold decrease) but mutation to glutamic acid almost completely blocked binding. Surprisingly, the conservative substitution of the cysteine sulfur by oxygen in the C104S mutant decreased the affinity by over 100-fold, almost as much as the introduction of a negative charge. The importance of the PRL3 catalytic cysteine was previously noted as its oxidation prevented binding to CNNM4 in immunoprecipitation and pulldown assays^7^.

Residues Leu108 and Arg138 are located outside of the catalytic site but make important contributions to binding (Fig. 3D,G). Substitution of Leu108 by alanine strongly decreased CNNM3 CBS-pair binding, likely by disrupting hydrophobic interactions with CNNM Pro427 and Tyr429 in the CBS extended loop. The interaction appears to be conserved across all CNNM and PRL proteins and likely plays a role in positioning the CNNM aspartic acid residue in the PRL catalytic site. Even more removed from the catalytic site, PRL3 Arg138 mediates one of the few interactions that does not involve the CBS extended loop. The arginine residue makes an electrostatic interaction with the CNNM3 aspartic acid residue, Asp396. Mutation of the arginine to either glutamic acid or alanine moderately decreased the affinity. The aspartic residue is conserved among vertebrate CNNM sequences but absent in CNNM proteins from *C*. *elegans*.

Mutation of Arg110 in the PRL3 catalytic site had the largest effect of any mutation and completely prevented binding in ITC experiments (Fig. 3E). The arginine is conserved across protein tyrosine phosphatase members and plays an essential role in positioning phosphatase substrates for catalysis^11^. In the PRL3•CNNM3 complex, the arginine similarly interacts electrostatically with CNNM3 Asp426. Substitution of the arginine with glutamic acid completely blocked binding. A second catalytic residue, Asp72, also plays a significant role in CNNM3 binding. Catalytically, the aspartic acid acts as a general acid contributing a proton^3^. In the PRL3•CNNM3 complex, the aspartic acid helps position the arginine and CBS extended loop.

The A111S mutation in PRL3 was the only one that did not decrease the affinity of binding (Fig. 3F). The alanine residue is typically serine or threonine in other protein tyrosine phosphatases and its substitution was previously shown to increase phosphatase activity^4^.

### Phosphatase activity assays

**Figure 4:**
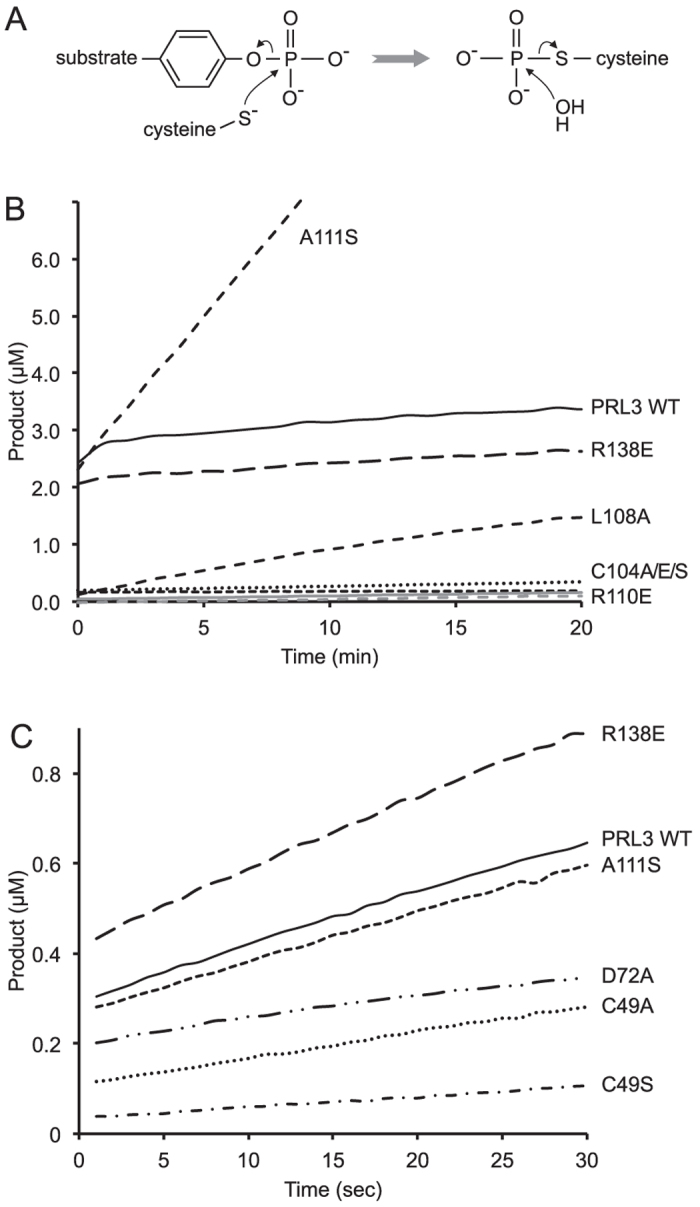
Phosphatase activity of PRL3 mutants. (**A**) Two-step catalytic cycle. Formation of a long-lived phosphocysteine catalytic intermediate leads to burst kinetics. (**B**) Time course of dephosphorylation of the fluorogenic substrate DiFMUP by wild-type PRL3 shows a fast kinetic step within the first minute of the assay followed by a slower steady-state rate limited by the hydrolysis of phosphocysteine. The A111S mutant shows the same burst phase as wild-type PRL3 but faster steady-state kinetics due to destabilization of the phosphocysteine intermediate. Mutation of cysteine 104 or arginine 110 completely inactivates the enzyme while the L108A mutant shows weak activity. The experimental dead time was approximately 2 minutes. (**C**) Time course over the first minute shows A111S has the same fast kinetics as the wild-type enyzme with various degrees of impairment for the other mutants. The delay between mixing and the first data point was roughly 20 seconds.

We next examined the effect of the mutations on PRL3 phosphatase activity (Fig. 4). Like other protein tyrosine phosphatases, PRLs have a two-step kinetic cycle, in which catalysis proceeds through a phosphocysteine intermediate^3^. In the case of PRL3, the phosphocysteine intermediate is extremely long-lived, giving rise to burst kinetics, characterized by a rapid step of dephosphorylation followed by a much slower steady-state rate^4^. Based on the steady-state rate, the lifetime of the phosphocysteine intermediate is on the order of 2 hours, in agreement with values from direct measurement of PRL2 phosphorylation^5^.

We observed a strong correlation between mutations that disrupt CNNM binding and phosphatase activity. As expected, mutation of the catalytic cysteine residue to alanine, serine or glutamic acid completely inactivated catalysis (Fig. 4B). Similarly the R110A mutant was completely inactive. The L108A mutant retained some residual kinetic activity, on the order of 3% of wild-type. As we showed previously^4^, mutation of alanine 111 to serine increased the steady-state rate of dephosphorylation 15-fold due to an increase in the hydrolysis of the phosphocysteine intermediate. Since the mutation does not affect the initial formation of phosphocysteine intermediate, it had no affect on the kinetics during the burst phase (Fig. 4C).

In order to characterize the initial burst kinetics, we modified the phosphatase assay by reducing the substrate concentration and delay before the first data points (Fig. 4C). This allowed us to quantify the activity of mutants which were only slightly affected. The most notable mutant was R138E which showed wild-type phosphatase activity but is moderately impaired in its affinity for CNNM3. The D72A, C49A and C49S mutants were somewhat impaired in their catalytic activity, in degrees that roughly corresponded to their modest impairment in CNNM3 binding. As observed for the catalytic cysteine, the conservative cysteine-to-serine mutant was more affected than the cysteine-to-alanine mutant.

### PRL3 phosphorylation

**Figure 5:**
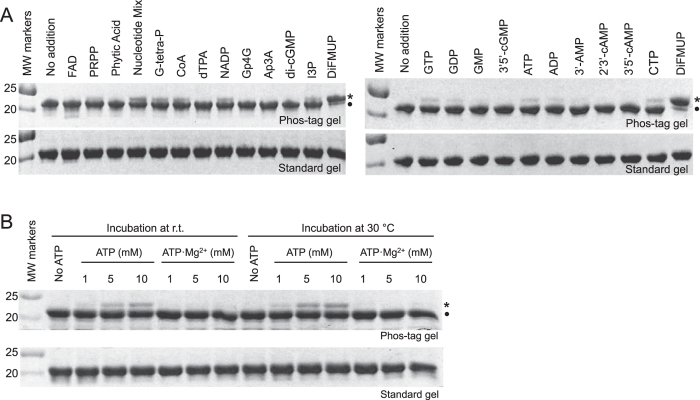
Phosphorylation of PRL3 by nucleotides and nucleotide analogs *in vitro*. (**A**) Indicated compounds were incubated with PRL3 and the phosphorylation of the catalytic cysteine detected by SDS-PAGE gels with or without the phos-tag reagent. The phosphorylated form of PRL-3 (*) migrates more slowly than the non-phosphorylated form (•) on the phos-tag gel. Compounds tested were FAD, phosphoribosyl pyrophosphate (PRPP), inositol hexakisphosphate (phytic acid), a mixture of nucleotides, guanosine 5’-tetraphosphate (G-tetra-P), coenzyme A (CoA), diethylenetriaminepentaacetic acid (dTPA), NADP, diguanosine tetraphosphate (Gp4G), diadenosine triphosphate (Ap3A), cyclic di-GMP (di-cGMP), inositol 1,4,5-trisphosphate (IP3), DiFMUP, GTP, GDP, GMP, 3’5’-cGMP, ATP, ADP, 3’-AMP, 2’3’-cAMP, 3’5’-cAMP, and CTP. (**B**) Magnesium prevents phosphorylation of PRL3 by ATP. Phos-tag gels show that purified PRL3 becomes partially phosphorylated in the presence of ATP but not ATP·Mg^2+^.

The stability of the phosphocysteine intermediate suggests that even poor substrates could lead to significant phosphorylation of the catalytic cysteine *in vivo*. We screened a collection of phosphorylated metabolites for their ability to phosphorylate PRL3 using phos-tag gels that separate the phosphorylated and unphosphorylated forms of the protein (Fig. 5A). Incubation of PRL3 with DiFMUP lead to essentially complete phosphorylation of PRL3. The small fraction of unphosphorylated protein could be due to the presence of oxidized PRL3 or spontaneous dephosphorylation prior to SDS-PAGE analysis.

While none of the metabolites tested showed activity comparable to DiFMUP, significant phosphorylation was observed with nucleotides and related compounds. The highest level of phosphorylation was 10 to 15% with nucleoside triphosphates. PRL3 did not show specificity beyond a requirement for diphosphate. We note that recombinant PRLs are significantly (up to 50%) cysteine phosphorylated when purified from *E*. *coli* ^5^, which suggests that the metabolites tested could be a significant source of phosphorylation in bacterially produced PRLs.

As endogenous levels of PRL cysteine phosphorylation in cultured mammalian cells varies in response to magnesium availability^5^, we asked if phosphorylation of PRL3 by ATP could act as a sensor of intracellular magnesium levels (Fig. 5B). The majority of cellular magnesium is bound to nucleotides and magnesium has been shown to be required for ATP binding to the CBS-pair domain of CNNM2^12^. Incubation of PRL3 with ATP lead to PRL3 phosphorylation in a concentration dependent fashion indicative of a *K*_*M*_ for ATP above 2 mM. In the presence of equal molar magnesium, ATP did not lead to PRL3 phosphorylation. The observation of magnesium-sensitive phosphorylation of PRL3 by nucleoside triphosphatase could be a mechanism for the changes in PRL phosphorylation observed in cultured cells upon magnesium depletion^5^.

## Discussion

The PRL family consists of three closely related proteins: PRL1, PRL2, and PRL3. They all contain a C-terminal prenylation site and a single catalytic domain of roughly 170 amino acids. The proteins share roughly 80% amino acid sequence identity overall and are highly similar in the composition and structure of their catalytic sites. All three PRLs have an unusual catalytic cycle which is limited by the extremely slow hydrolysis of a phosphocysteine catalytic intermediate. Crystal structures alone or in complexes have been reported for PRL1^13, 14^ and PRL2^5^ and solution NMR structures reported for PRL3^4, 15^. The structure of PRL3 with the CNNM3 CBS-pair domain is the first crystal structure of PRL3. As might be expected from their high degree of sequence conservation, all three PRL phosphatases have been shown to bind CNNM proteins^7–9^.

The four CNNM proteins, CNNM1-4, are membrane proteins and play a role in magnesium transport^16, 17^. The proteins are larger (700–950 amino acids) and more diverse than PRLs but show strong sequence conservation in their CBS-pair domains and the loop that binds PRL proteins. CBS-pair domains are found in many different enzymes such as AMP kinase or the bacterial magnesium transporter MgtE^18^. In addition to the CBS-pair domains, CNNM proteins have a extracellular region, a conserved transmembrane domain, and a putative cyclic nucleotide binding domain adjacent to the CBS-pair domain. The CNNM CBS-pair domains bind adenine nucleotides and are thought to play a role in magnesium sensing^12^. Crystal structures have been determined for the CBS-pair domains from CNNM2, CNNM3 and CNNM4^5, 9, 10^ alone or in complexes with PRL phosphatases.

We used ITC experiments to quantify the affinity and observed low nanomolar affinity for PRL2 and PRL3 binding to the CNNM3 CBS-pair domain (Fig. 3). Curiously, the reported affinity of PRL1 for the CNNM2 domain is 60-fold weaker (0.64 *μ*M) although all three PRLs showed similar binding by gel filtration^9^. We previously reported slightly weaker affinity for the PRL2-CNNM3 interaction, which we attribute to the presence of some PRL oxidation^5^. The catalytic cysteine has a strong tendency to form a disulfide and requires high concentrations of DTT for complete reduction^4^. The presence of oxidized protein is most likely responsible for the weaker affinity reported for the PRL1-CNNM2 interaction^9^.

The availability of multiple structures of PRL•CNNM complexes allows a detailed dissection of the effects of cysteine oxidation. Disulfide bond formation strongly decreases the affinity of CNNM binding through two distinct mechanisms. Firstly, as a thiol, the sulfur of the catalytic cysteine contacts the CNNM aspartic acid residue. Crystal structures of PRL3 (C104A), and the oxidized forms of PRL2 and PRL1 all show a deep pocket which is closed off in the complex with reduced PRL2 (Fig. 1 and 2A). The size and polarizability of sulfur cannot be replaced by oxygen and indeed the C104S mutant has lower affinity that the C104A mutant. Removing the sulfur is better than replacing it with oxygen. Secondly, formation of the disulfide turns the loop containing the catalytic cysteine so that the following alanine residue flips to displace the incoming CNNM aspartic acid (Fig. 2B).

A misconception about PRL phosphatases is that they are inactive due to the absence of a good substrate. The low kinetic rate is due to the slow hydrolysis of the phosphorylated form of the enzyme and not substrate dependent. The initial burst rate is several hundred times faster than the steady-state rate and can easily be overlooked in *in vitro* phosphatase assays. The burst is limited to one turnover so the amount of product generated is relatively small and can be mistaken for an offset error in the detector. PRL phosphatases readily oxidized even in the presence of DTT which can further reduce their activity both *in vitro* and *in vivo*. High concentrations of freshly prepared DTT are required to fully reduce the PRL3 catalytic cysteine^4, 6^. As neither PRL oxidation nor its dephosphorylation rate are dependent on the substrate, there is no basis for believing PRLs will show higher steady-state activity if a better substrate is found.

Remarkably, the most sensitive method for detecting PRL3 phosphatase activity is to observe PRL3 phosphorylation rather than product generation. The long lifetime of the phosphocysteine intermediate allows the activity of even very slow substrates to be measured. We observed significant phosphorylation of PRL3 by physiological (mM) concentrations of nucleotide triphosphates (Fig. 5). This explains our observation that PRL proteins are partially phosphorylated in bacterial cells which are very unlikely to harbor physiological phosphoprotein or phospholipid substrates^5^. Our observation of magnesium inhibition is highly evocative for explaining changes in PRL cysteine phosphorylation upon magnesium withdrawal in mammalian cells; however, the effect is opposite of that predicted by a simple model. We observed magnesium inhibition of cysteine phosphorylation while the cells showed less phosphorylation in the absence of magnesium in the culture medium^5^.

A key question relating to the role of PRLs in promoting cancer is the relative importance of PRL phosphatase activity versus CNNM binding. In cellular assays, the binding of PRL3 to CNNM4 inhibits CNNM-dependent magnesium efflux^12^. The inhibition is dependent on the CNNM4 aspartic acid in the PRL-binding loop^5^ but the mechanism of oncogenicity of PRLs remains undecided. Studies of a mouse model of metastatic disease observed that the PRL3 C104S mutant was inactive in promoting metastases^7^; however, the serine substitution blocks both binding and activity and so doesn’t distinguish between them (Figs 3 and 5). That same study also showed that PRL3 C49S was inactive in the cancer model^7^. Again our *in vitro* studies show the C49S mutant has comparable (although more minor) deficits in CNNM3 binding and phosphatase activity. An additional complication is that cysteine 49 is thought to have a protective role against PRL oxidative damage so the consequences of its mutation in cancer cells are unclear^6^.

In contrast, we observed that the R138E mutant is strongly affected in CNNM binding but shows wild-type phosphatase activity. This offers the possibility of separating PRL phosphatase activity versus CNNM binding in cancer models. Testing the PRL3 A111S mutant should also be informative since the phosphocysteine intermediate is significantly less stable. The PRL3 A111S mutant protein should more strongly associate with CNNM proteins which may make it more oncogenic than wild-type PRL3.

## Methods

### Expression and purification of proteins

Wild-type PRL2 phosphatase (residues 1–167) and a triple mutant (C95A, C96A, C119A) additionally missing the C-terminal prenylation site were expressed and purified as a His-tag fusion proteins as described previously^5^. Human phosphatase PRL3, amino acids 1–169, was inserted into the pET15b vector (Novagen Inc., Madison, WI) and expressed and purified as a 6-His tag fusion protein with a thrombin cleavage site leaving an N-terminal extension of Gly-Ser-His. PRL3 point mutants were generated with QuikChange Lighting Site-Directed Mutagenesis Kit (Agilent Technologies) and confirmed by DNA sequencing and mass spectrometry. The CBS-pair domain of human CNNM3 was prepared as previously described^5^. The murine CNNM3 CBS-pair domain (residues 316–458) was cloned into the pGEX-6P-1 vector to produce a GST-tagged protein. The GST tags were removed for crystallization and the proteins were exchanged into a buffer containing 20 mM HEPES, 100 mM NaCl, 5 mM tris(2-carboxyethyl)phosphine, pH 7.0.

### Crystallization and data collection

Crystals of the PRL3 C104A•CNNM3 CBS-pair domain complex were obtained using hanging drops at 293 K with 0.8 *μ*l drops of a 1:1 mixture of the two proteins (1 mg/ml) in 20 mM HEPES pH 7.0, 100 mM NaCl, 5 mM TCEP buffer equilibrated over 0.064 M sodium citrate, 15% (w/v) PEG5000 MME, 0.1 M HEPES pH 7.0. Crystals of the PRL-2•CNNM3 CBS-pair domain complexes were obtained using 0.7 *μ*l drops of a 1:1 mixture of the proteins (5 mg/ml) equilibrated over 0.54 M sodium citrate and 0.1 M sodium acetate pH 5. Nucleotide-bound crystals were obtained by adding 2.5 mM AMP and 10 mM ATP to the crystallization drop. The cryoprotection solution contained crystallization condition supplemented with 30% (v/v) glycerol. Diffraction data were collected at wavelength 0.9770 Å on beamline F1 at the Cornell High-Energy Synchrotron Source (Suppl. Table S1). Data processing and scaling were performed with HKL-2000^19^.

### Structure solution and refinement

The starting phases for the structures were obtained using molecular replacement with the PRL2•CBS-pair complex (PDB entry 5K22) in Phaser^20^. The resulting models were extended manually with the help of the program Coot^21^ and improved by several cycles of refinement using PHENIX^22^. The final models have good stereochemistry (Suppl. Table S1). Figures were produced using PyMOL (http://www.pymol.org).

### Isothermal titration calorimetry

ITC experiments were performed on a MicroCal VP-ITC titration calorimeter (Malvern Instruments Ltd). The syringe was loaded with 160 *μ*M PRL2 or PRL3 while the sample cell contained 16 *μ*M CNNM3 CBS-pair domain as a GST fusion. All experiments were carried out at 22 °C with 30 injections of 10 *μ*l for a total of 2 h with stirring at 310 rpm. Results were analyzed using ORIGIN software (MicroCal) and fitted to a binding model with a single set of identical sites.

### Phosphatase assays

Phosphatase activity was measured with a fluorogenic substrate, 6,8-difluoro-4-methylumbelliferyl phosphate (DiFMUP) purchased from Molecular Probes, Thermo Fisher Scientific, as previously described^5^. The reaction buffer contained 20 mM HEPES, 100 mM NaCl, 5 mM TECEP or 10 mM 1,4-dithiothreitol (DTT), pH 7.0. For measurements of the steady-state rate, the final concentrations were 3 *μ*M protein and 100 *μ*M DiFMUP in a final volume of 50 *μ*l. Control sample was the corresponding reaction buffer. For burst measurements, the DiFMUP concentration was reduced to 30 *μ*M. The fluorescence of product was detected using excitation at 360 nm and emission at 455 nm (SpectraMax M5e, Molecular Devices, LLC.) at room temperature. The velocities against DiFMUP were calculated from measurements of free DiFMU that which was derived from standard curve of coumarine substrate from 0 to 200 *μ*M concentrations. The PRL3 concentrations were measured by absorbance at 280 nm using an estimated extinction coefficient of 19940 M^−1^ cm^−1^.

### Screening potential substrates for PRL3

Samples of 0.3 mg/ml PRL3 in 20 mM HEPES, 100 mM NaCl, 10 mM DTT, pH 7.0 buffer were treated with potential substrates (nucleotides and nucleotide analogs) at 100 *μ*M final concentration and incubating for 1 hour at room temperature. Reactions were stopped with the SDS-PAGE loading dye containing 10 mM DTT for SDS-PAGE analysis. Aliquots of 8 *μ*l of the reactions were separately loaded on 17.5% Tris-tricine gels with and without 5.0 mM phostag reagent (Wako Chemicals) and 10 mM MnCl_2_ according to ref. 23. Gels were visualized with Coomassie Brilliant Blue staining.

### PRL3 phosphorylation assay with ATP and ATP·Mg^2+^

To further investigate the effect of ATP or ATP·Mg^2+^ on PRL3-wt phosphorylation, we carried out each 43 *μ*M PRL3-wt with different ATP concentrations and ATP·Mg^2+^ (molar ratio = 1:1) concentrations in a reaction volume 25 *μ*l of 20 mM HEPES, 100 mM NaCl, 10 mM DTT, pH 7.0 buffer. Samples were incubated at 30 °C and room temperature. The reactions were terminated by the addition of gel loading buffer and analyzed by Phos-tag SDS-PAGE as described above.

### Data availability

The atomic coordinates and structure factors have been deposited in the Protein Data Bank, Research Collaboratory for Structural Bioinformatics, Rutgers University, New Brunswick, NJ (http://www.rcsb.org/).

## Electronic supplementary material

Supplementary information
