# Erratum: PRL3 phosphatase active site is required for binding the putative magnesium transporter CNNM3

**DOI:** 10.1038/s41598-017-04855-7

**Published:** 2017-07-21

**Authors:** Huizhi Zhang, Guennadi Kozlov, Xinlu Li, Howie Wu, Irina Gulerez, Kalle Gehring

**Affiliations:** 1 Department of Biochemistry, McGill University, Montreal, Quebec Canada; 2 School of Chemical Engineering, Northwest University, Xi’an, 710069 China

*Scientific Reports* **7**:48; doi:10.1038/s41598-017-00147-2; Article published online 03 March 2017

In the original HTML version of this Article, Kalle Gehring was incorrectly listed as being affiliated with ‘School of Chemical Engineering, Northwest University, Xi’an, 710069, China’. The correct affiliation is listed below:

Department of Biochemistry, McGill University, Montreal, Quebec, Canada.

This error has now been corrected in the HTML version; the PDF version was correct at the time of publication.

