# Supplementary material for: PRL3 phosphatase active site is required for binding the putative magnesium transporter CNNM3
Source: Sci Rep. 2017 Mar 3;7:48. doi: 10.1038/s41598-017-00147-2 (PMC5427921; doi:10.1038/s41598-017-00147-2)
Supplement: Supplementary file 1 — Supplementary information [file 41598_2017_147_MOESM1_ESM.pdf]

PRL3 phosphatase active site is required for binding the putative magnesium transporter CNNM3

Huizhi Zhang [1,2,+], Guennadi Kozlov [1,+], Xinlu Li [1], Howie Wu [1], Irina Gulerez [1], and Kalle Gehring [2,*]

[1]Department of Department of Biochemistry, McGill University, Montreal, Quebec, Canada
[2]Present address: School of Chemical Engineering, Northwest University, Xi'an 710069, China
[*]kalle.gehring@mcgill.ca
[+]these authors contributed equally to this work

Supplemental Material

Table S1. Data Collection and Refinement Statistics

Accession number	5TSR	5K23	5K24	5K25
Data collection	PRL3-C104A • hCNNM3	PRL2 • hCNNM3	PRL2 • mCNNM3	ATP • PRL2 • hCNNM3
Space group	C2	I222	C2	I222
Cell dimensions				
a, b, c (Å)	155.3, 125.1, 52.0	51.2, 124.5, 159.0	154.2, 52.1, 102.9	52.1, 124.4, 164.3
α, β, γ ( )	90, 102.2, 90	90, 90, 90	90, 106.0, 90	90, 90, 90
Resolution (Å)	50-3.20 (3.26-3.20) [1]	50-2.95 (3.00-2.95)	50-3.1 (3.15-3.1)	50-3.05 (3.1-3.05)
Rsym	0.106 (0.534)	0.047 (0.444)	0.078 (2.8)	0.066 (0.476)
I / σI	10.5 (1.5)	24.3 (3.2)	33.9 (2.8)	26.6 (4.5)
Completeness (%)	96.6 (99.6)	99.9 (100)	99.7 (100)	99.8 (100)
Redundancy	3.3 (3.0)	4.8 (4.9)	4.6 (4.7)	5.6 (5.7)
Refinement				
Resolution (Å)	27.7 - 3.19	49.0 - 2.96	98.9 - 3.10	49.7 - 3.05
No. reflections	15609	10440	13689	10010
Rwork / Rfree	0.239/0.287	0.205/0.264	0.252/0.303	0.232/0.283
No. atoms				
Protein	4764	2401	4509	2418
Nucleotide			27	
Water				3
B-factors				
Protein	113.1	69.8	63.9	92.3
Nucleotide				96.6
Water				61.7
R.m.s deviations				
Bond lengths (Å)	0.009	0.011	0.004	0.004
Bond angles ( )	1.57	1.8	0.8	0.8
Ramachandran statistics (%)				
Most favored regions	92.5	95.4	91.9	92.1
Additional allowed regions	6.2	3.6	6.9	5.9
Disallowed regions	1.3	1.0	1.2	2.0

[1] Highest resolution shell.

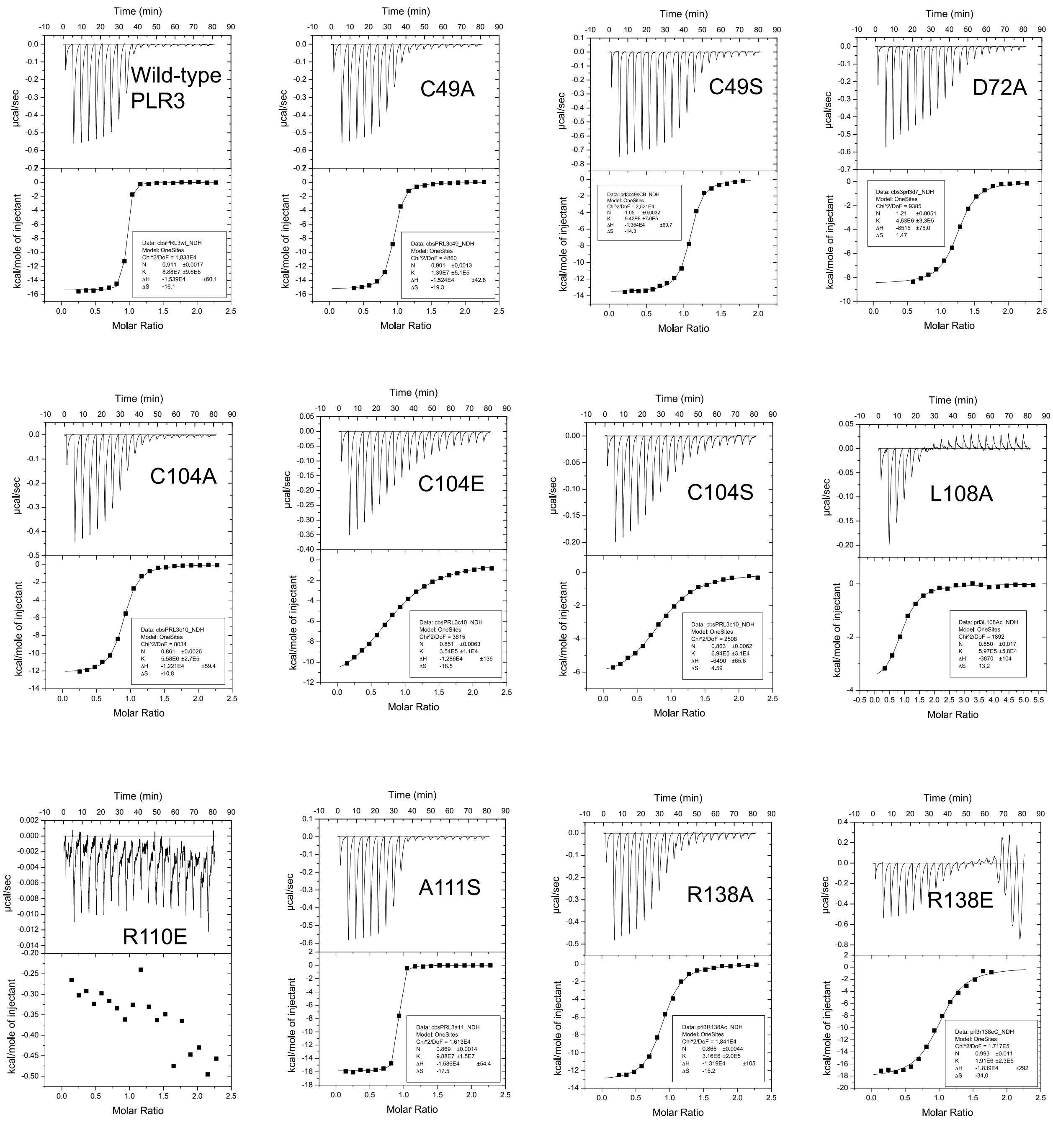

Figure S1. Isothermal titration calorimetry traces of wild-type and mutant PRL3 proteins binding the CNNM3 CBS-pair domain. Thermograms are shown above the fittings of the heat released. PRL3 R110E showed no heat release.

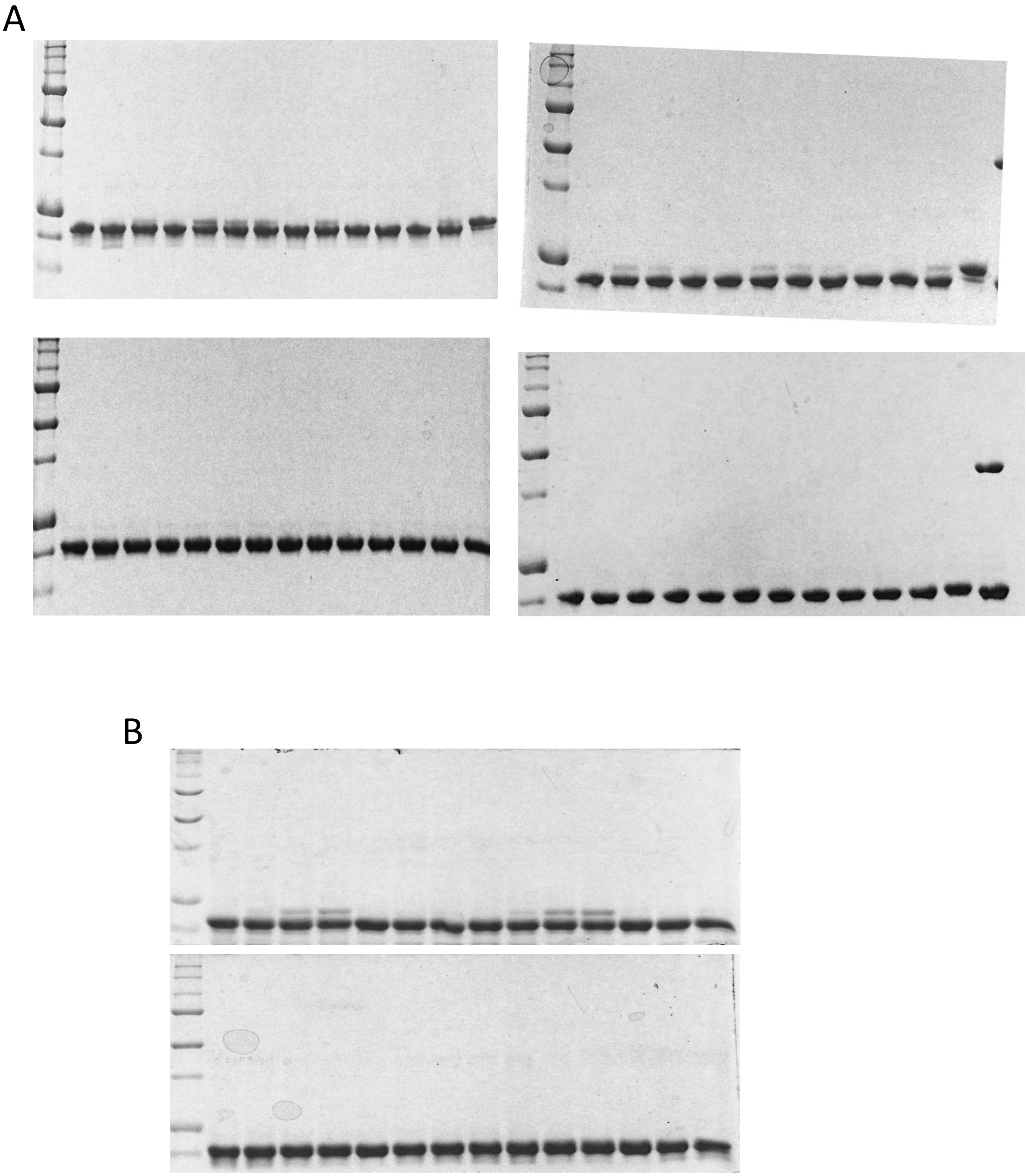

Figure S2. Uncropped Coomassie-stained SDS-PAGE gels from Figure 5. A, Screening of different compounds. B, Inhibition by magnesium.
